# Regional Disparities in Mortality Trends for Hypertensive Heart Disease With and Without Heart Failure in the United States: A Negative Binomial Modeling and Forecasting Analysis

**DOI:** 10.7759/cureus.98690

**Published:** 2025-12-08

**Authors:** Hunter W Brady, Rachel LaRaut, Bridget Baur, Madeline Dorminy, Gregory Keagy

**Affiliations:** 1 Department of Medicine, Lincoln Memorial University DeBusk College of Osteopathic Medicine, Knoxville, USA; 2 Department of Medical Education, Lincoln Memorial University DeBusk College of Osteopathic Medicine, Knoxville, USA; 3 Department of Surgery, Lincoln Memorial University DeBusk College of Osteopathic Medicine, Knoxville, USA

**Keywords:** annual percent change, cardiovascular mortality, cdc wonder, heart failure, hypertensive heart disease, negative binomial regression, regional disparities

## Abstract

Introduction

Hypertensive heart disease (HHD) is a major contributor to cardiovascular mortality worldwide. In the United States, HHD mortality has risen in recent decades despite advances in antihypertensive therapy and cardiovascular care. This study aimed to evaluate the trends in age-adjusted mortality from HHD with and without heart failure across the four U.S. census regions (Northeast, Midwest, South, and West).

Methods

National mortality data for HHD with and without heart failure as the underlying cause of death from 2018-2023 were obtained from the Centers for Disease Control and Prevention (CDC) Wide-ranging Online Data for Epidemiologic Research (WONDER) database and stratified by U.S. census region. A negative binomial regression model was used to estimate annual percent changes (APC) in age-adjusted mortality rates for HHD with heart failure and HHD without heart failure across U.S. census regions. Age-adjusted mortality rates are standardized to the U.S. 2000 standard population and reported per 100,000 population. APC was derived with Wald 95% confidence intervals (CIs) and p-values. Pairwise slope contrasts were conducted to assess regional differences. Forecasting through 2030 was performed using gamma regression modeling, which extrapolates trends estimated from the observed 2018-2023 data.

Results

For HHD with heart failure, mortality increased significantly across all regions, with the steepest rise in the Midwest (+10.0%/year, 95% CI: 9.16-10.85; p = 2.1×10⁻¹³⁰) and South (+10.7%/year, 95% CI: 8.98-12.37, p = 2.46×10⁻³⁸). Pairwise contrasts confirmed steeper slopes in the Midwest and South vs. the Northeast (p < 1×10⁻⁷ and p < 1×10⁻¹⁰, respectively), and the South exceeding the West (p = 0.018). For HHD without heart failure, increases in APC were observed in the Midwest (+7.9%/year, 95% CI: 5.01-10.99, p = 6.1×10⁻⁸), South (+5.8%/year, 95% CI: 3.37-8.26 p = 1.81×10⁻⁶), and West (+6.1%/ year, 95% CI: 3.44-8.86, p = 5.15×10⁻⁶), while the Northeast showed no significant trend (1.7%/year, 95% CI: −2.67 to 6.32, p = 0.45 ). Pairwise slope analysis revealed that the Midwest was the only region that differed significantly from the Northeast (p = 0.049). Forecasted 2030 mortality rates reinforced these findings, with HHD with heart failure projected to be highest in the South (17.83 per 100,000 people; 95% CI: 14.64-21.73) and HHD without heart failure highest in the South and Midwest.

Conclusions

Mortality from HHD with heart failure is rapidly increasing nationwide and projected to rise further by 2030. There is a disproportionate burden in the Midwest and South, highlighting geographic disparities in these regions. In contrast, HHD without heart failure shows moderate increases, primarily driven by trends in the Midwest, and is also projected to grow by 2030. These results highlight the urgent need for region-specific interventions to mitigate hypertension and cardiovascular risk factors.

## Introduction

Hypertensive heart disease (HHD) is characterized by structural and functional alterations of the left ventricle, left atrium, and coronary circulation resulting from prolonged hypertension [1]. The chronic pressure overload leads to left ventricular hypertrophy, which can eventually progress to heart failure [1]. Both HHD and heart failure independently remain major contributors to cardiovascular morbidity and mortality in the United States [2]. Hypertension is the primary cause of HHD; therefore, blood pressure management significantly influences the prognosis [3]. Unfortunately, the effective management and prevention of HHD face numerous challenges, including difficulties in access to primary healthcare, limited insurance coverage, limited affordable medication therapies, and inadequate screening practices, all of which contribute to the rising mortality of HHD [4-6]. 

While several studies have shown a rise in mortality in the United States due to HHD over the years, few have explored regional trends or mortality patterns specifically in relation to heart failure [5]. Variations in healthcare accessibility, socioeconomic status, and comorbidities contribute to differing regional trends in cardiovascular mortality, and hence these differences need to be explored further [5]. Overall, the global prevalence of HHD increased between 1990 and 2021, with metropolitan areas generally experiencing higher rates than non-metropolitan regions [2,5]. However, few studies have specifically examined regional HHD mortality while explicitly distinguishing between cases with and without heart failure as a comorbidity.

Regional variations in cardiovascular outcomes must be evaluated to identify health disparities and tailor public health interventions to address specific socioeconomic conditions, healthcare access, and insurance coverage [7]. Variations in the prevalence of modifiable risk factors, including hypertension, diabetes, smoking, physical inactivity, and dietary patterns, contribute to varying disease burdens across regions. Distinguishing between HHD cases with and without heart failure may offer valuable insights, since heart failure substantially elevates mortality risk compared to HHD alone [6]. Therefore, the combined impact of HHD and heart failure on mortality warrants further investigation.

The objective of this study was to quantify regional trends in age-adjusted mortality from HHD with and without heart failure across U.S. census regions (Northeast, Midwest, South, and West) from 2018 to 2023 and to project these trends through 2030. Distinguishing mortality with and without concurrent heart failure is clinically important, as heart failure substantially increases mortality risk, and incorporating short-term forecasting provides predictive context to supplement the primarily descriptive and comparative aims of this analysis.

## Materials and methods

Study design and data source

This was a retrospective, population-based study utilizing publicly available data reported by the Centers for Disease Control and Prevention (CDC) Wide-ranging Online Data for Epidemiologic Research (WONDER) database [8]. CDC WONDER records death certificate information submitted by all 50 U.S. states and the District of Columbia, with causes of death coded using the International Classification of Diseases (ICD). Age-adjusted mortality rates are standardized to the U.S. 2000 standard population. Data extraction was performed using CDC WONDER’s mortality underlying cause-of-death system, and variables obtained included year of death, U.S. census region (Northeast, Midwest, South, and West), regional population estimates, number of deaths, and age-adjusted mortality rates. Mortality data were stratified according to the four U.S. Census regions.

Study population and eligibility criteria

The study included U.S. resident deaths from 2018-2023 where the underlying cause of death was coded as HHD with heart failure (I11.0) or without heart failure (I11.9). Only the underlying cause-of-death field was used; multiple-cause fields were not included. Non-U.S. residents and deaths lacking ICD-10 codes I11.0 and I11.9 were excluded. Instances in which CDC WONDER suppressed mortality counts due to small cell sizes were excluded because of the inability for age-adjusted rates to be reliably estimated; however, no suppressed cells were present in this dataset.

Statistical analysis

Negative binomial regression models were applied to the CDC WONDER age-adjusted mortality rates (per 100,000 population), which are pre-standardized to the 2000 U.S. population. The year was treated as a continuous predictor without centering or scaling. A negative binomial model was selected to account for overdispersion in the mortality counts. Annual percent change (APC) was derived as \text{APC}={e^{\beta\text{_year}}-1}\times 100\. Wald 95% confidence intervals (CI) and p-values were calculated for APC. Because only six annual observations were available, segmented trend-detection methods such as Joinpoint regression were not applied, as the limited time span may provide insufficient power to detect meaningful change-points. All predictors (census region and ICD-10 code categories) were retained in the models regardless of statistical significance, to allow for regional comparability.

Pairwise slope contrasts were conducted to test for differences in APC between regions with calculated 95% CI and p-values. Forecasting of predicted age-adjusted rates through 2030 was performed using gamma regression modeling. Models were fit with age-adjusted mortality rates as the outcome, specifying a log link and including year as the sole predictor. All analyses were conducted in R (version 4.5.1, R Foundation for Statistical Computing, Vienna, Austria) using RStudio (version 2025.05.1+513, Posit Software, PBC). Base statistical code was generated with support from a digital language-assistance tool, and all analyses were subsequently reviewed and validated by the study’s data analyst to ensure accuracy, consistency, and error-free execution [9]. 

## Results

Across the study period of 2018-2023, 216,681 deaths were identified by the CDC WONDER database, with 65,397 deaths attributed to HHD with heart failure and 151,284 deaths to HHD without heart failure. Populations within census regions ranged from 56 to 130 million. In 2018, age-adjusted rates for HHD with heart failure ranged from 3.9 in the Northeast to 6.0 in the West per 100,000 people, while rates for HHD without heart failure ranged from 6.1 in the West to 10.1 in the South per 100,000 people. By the end of the study period, it was observed that the highest age-adjusted mortality was in the South for both subgroups, with 8.5 per 100,000 for HHD with heart failure and 13.0 per 100,000 for HHD without heart failure (Table 1).

**Table 1:** Age-adjusted mortality rates for HHD with and without HF by U.S. census region: 2018 vs. 2023 HHD: hypertensive heart disease; HF: heart failure

Census region	2018 (I11.0, with HF)	2023 (I11.0, with HF)	2018 (I11.9, without HF)	2023 (I11.9, without HF)
Northeast	3.9	5.0	7.0	7.3
Midwest	4.4	7.3	7.0	9.6
South	5.3	8.5	10.1	13.0
West	6.0	8.6	6.1	7.5

Annual percentage change

**Figure 1:**
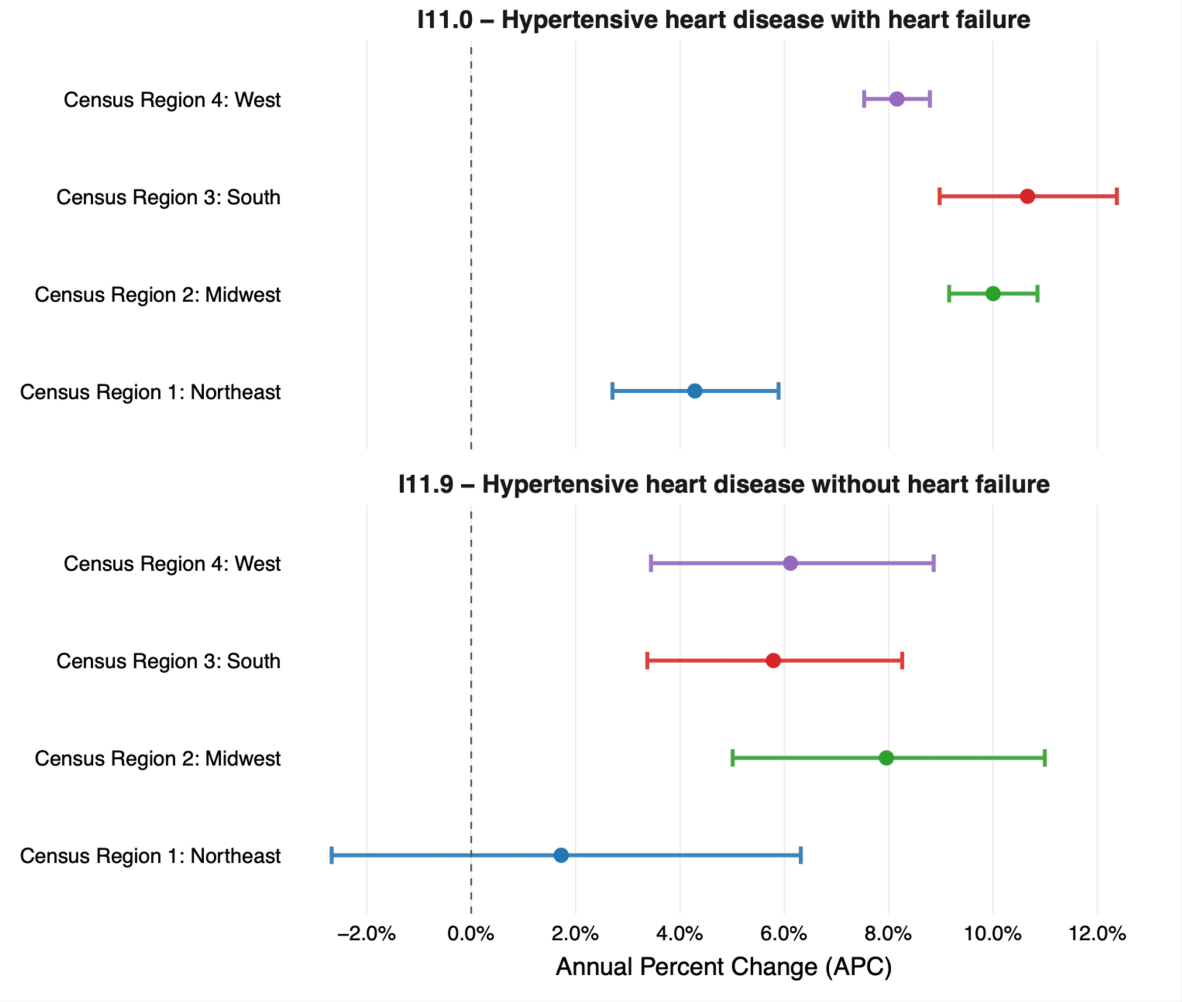
Annual percentage change in HHD with and without HF for all four US census regions from 2018 to 2023 HHD: hypertensive heart disease; HF: heart failure

From 2018 to 2023, mortality from HHD increased significantly across most U.S. census regions, with notable differences by region and by the presence of heart failure (Figure 1). For HHD with heart failure (ICD-10 I11.0), the Northeast experienced the slowest rise annually, with an APC of 4.3% (95% CI: 2.7-5.9; p < 0.001). While the Midwest and South showed the steepest increases at 10.0% (95% CI: 9.2-10.9; p < 0.001) and 10.7% (95% CI: 9.0-12.4; p < 0.001), respectively. The West exhibited a rise of 8.2% annually (95% CI: 7.5-8.8; p < 0.001).

For HHD without heart failure (ICD-10 I11.9), trends were more modest than with heart failure. The Northeast demonstrated no significant change with an APC of 1.7% (95% CI: −2.7 to 6.3; p = 0.45). This increase in the Northeast was not statistically significant, indicating that there is insufficient evidence to conclude that the observed APC reflects anything other than random variation. The Midwest (7.9%; 95% CI: 5.0-11.0; p < 0.001), South (5.8%; 95% CI: 3.4-8.3; p < 0.001), and West (6.1%; 95% CI: 3.4-8.9; p < 0.001) demonstrated significant upward trends in APC. Detailed APC values, confidence intervals, rate ratios, and regression coefficients for all regions and ICD categories are provided in Table 2.

**Table 2:** APC in HHD mortality by U.S. census region (2018-2023) APC: annual percentage change; HHD: hypertensive heart disease; ICD: International Classification of Diseases

ICD	Region	APC (%)	95% CI	Rate ratio	β_year	SE
I11.0	Northeast	4.28	2.95–5.62	1.0428	0.0419	0.0065
I11.0	Midwest	9.99	8.66–11.33	1.0999	0.0952	0.0062
I11.0	South	10.64	9.41–11.87	1.1064	0.1011	0.0057
I11.0	West	8.16	6.91–9.43	1.0816	0.0784	0.006
I11.9	Northeast	1.72	–1.41–4.96	1.0172	0.0171	0.016
I11.9	Midwest	7.96	4.65–11.38	1.0796	0.0766	0.0159
I11.9	South	5.79	2.58–9.11	1.0579	0.0563	0.0158
I11.9	West	6.12	2.86–9.49	1.0612	0.0594	0.0159

Pairwise slope contrasts 

**Figure 2:**
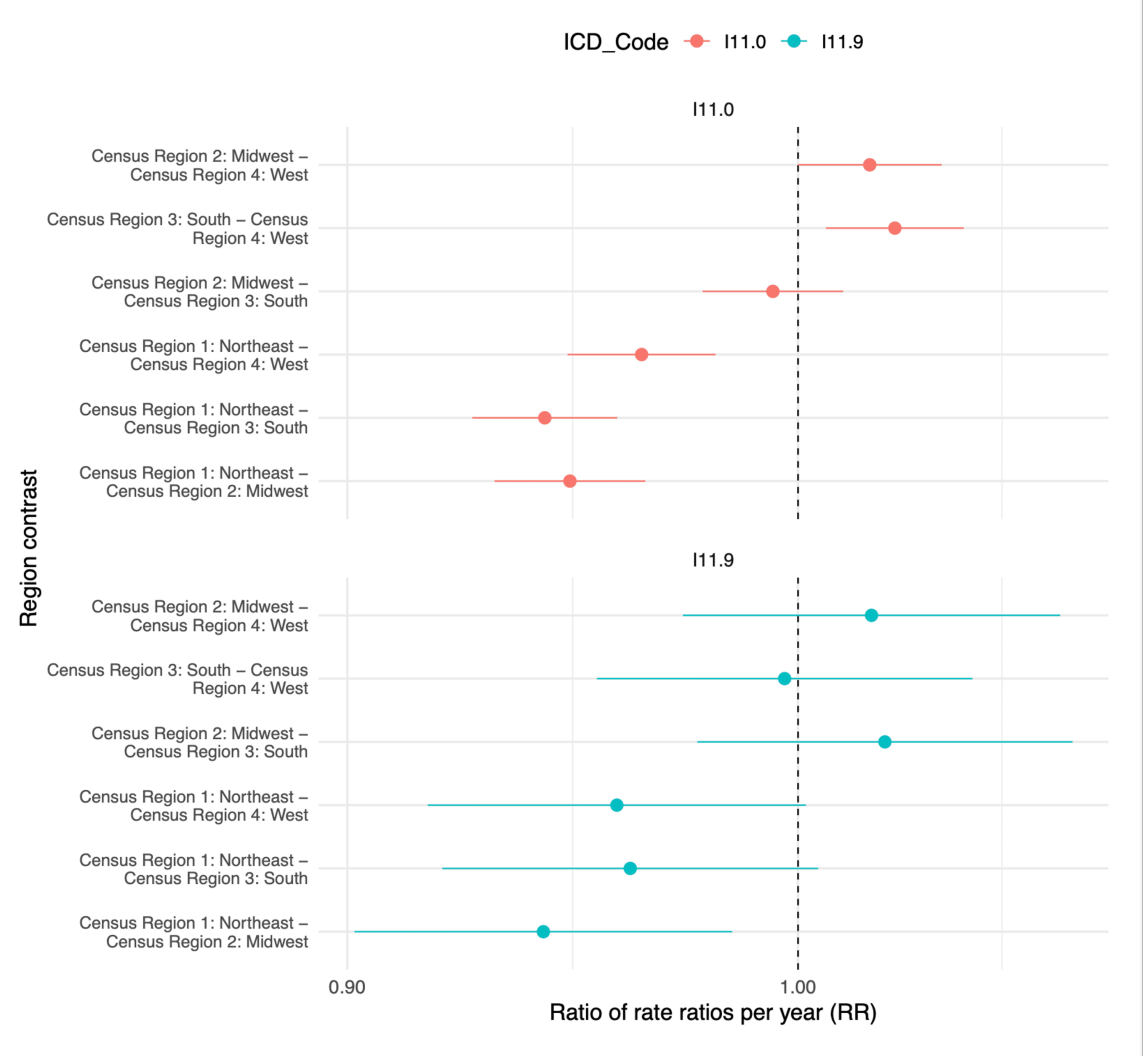
Pairwise census-region contrast analysis in annual rate ratio or HHD mortality from 2018 to 2023 HHD: hypertensive heart disease; ICD: International Classification of Diseases

Pairwise slope contrasts demonstrated that regional disparities in mortality trends exist, particularly in the Midwest and South (Figure 2). For HHD with heart failure (ICD-10 I11.0), compared to the Northeast, the Midwest (relative rate ratio per year (RRR) = 0.948, p < 0.001) and South (RRR = 0.943, p < 0.001) demonstrated significantly steeper increases. The West also showed a faster rise than the Northeast (RRR = 0.964, p < 0.001). No significant difference was detected between the Midwest and South (RRR = 0.994, p = 0.48). In contrast, the South experienced a significantly sharper increase than the West (RRR = 1.023, p = 0.018).

For HHD without heart failure (ICD-10 I11.9), the Midwest showed a significantly faster increase than the Northeast (RRR = 0.942, p = 0.049). All other comparisons - including the South versus Northeast (RRR = 0.962, p = 0.32), West versus Northeast (RRR = 0.959, p = 0.30), Midwest versus South (RRR = 1.021, p = 1.00), Midwest versus West (RRR = 1.017, p = 1.00), and South versus West (RRR = 0.997, p = 1.00) - were not statistically significant.

Gamma regression 2030 forecasting

**Figure 3:**
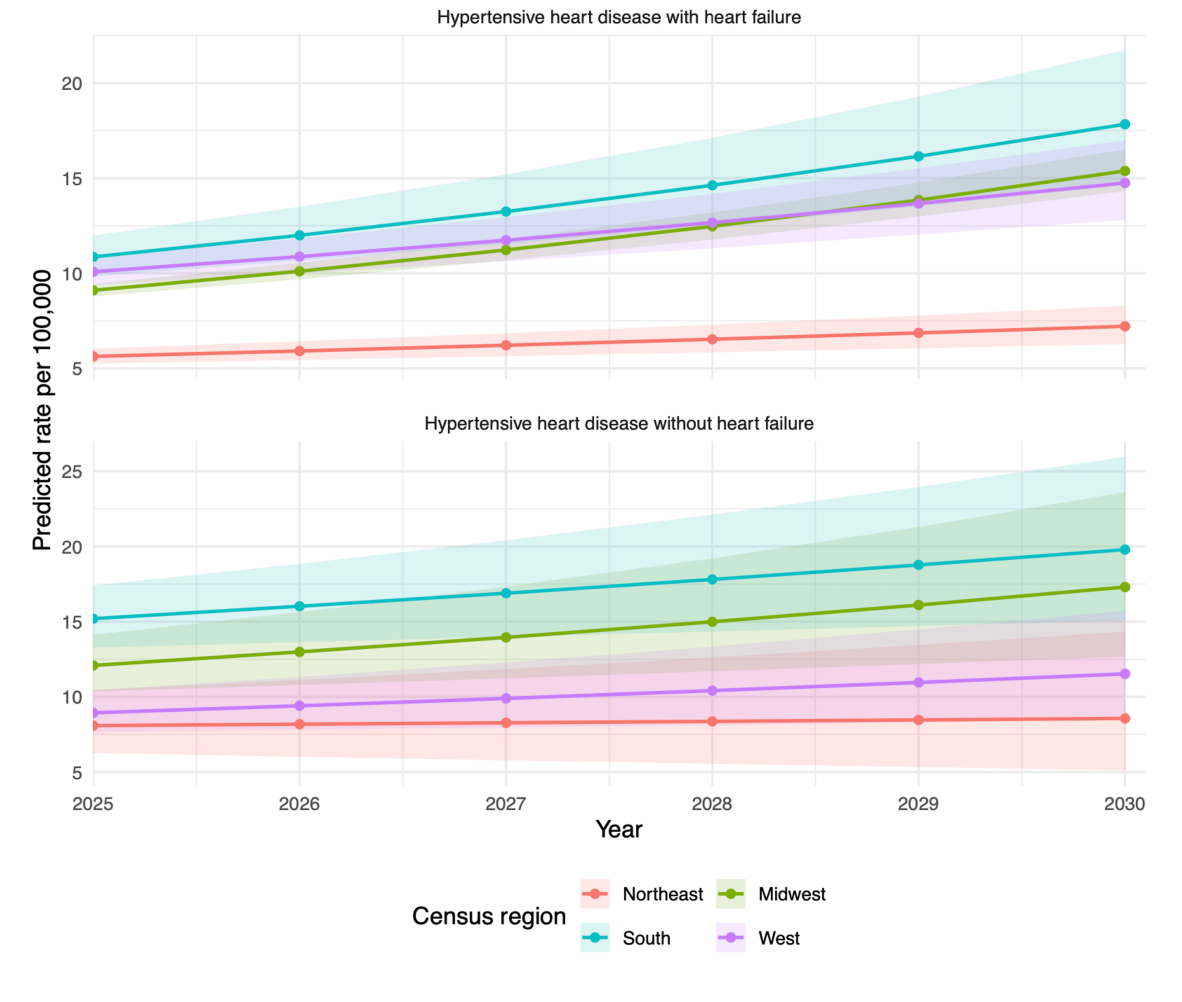
Age-adjusted mortality rate gamma regression model for projected mortality from HHD between 2025 and 2030 HHD: hypertensive heart disease

Exploratory gamma regression projections suggest that disparities may widen through 2030, with the South and Midwest maintaining the steepest increases (Figure 3). For HHD with heart failure (ICD-10 I11.0), age-adjusted mortality rates in the South are projected to increase from 5.3 in 2018 to 17.8 per 100,000 in 2030. The Midwest is projected to rise from 4.4 to 15.4 per 100,000. Additionally, rates in the West are projected to increase from 6.0 to 14.7 per 100,000 and in the Northeast from 3.9 to 7.2 per 100,000.

For HHD without heart failure (ICD-10 I11.9), rates in the Northeast and Midwest are expected to rise from 7.0 in 2018 to 8.6 and 17.3 per 100,000 people, respectively, in 2030. The rates in the South are expected to rise from 10.1 to 19.8 per 100,000 people by 2030, and those in the West from 6.1 to 11.5 per 100,000 people.

## Discussion

This study demonstrates a significant rise in mortality from HHD in the United States from 2018 to 2023, with a notably sharper rise in cases involving heart failure (ICD-10 I11.0) compared with HHD without heart failure (ICD-10 I11.9). Regional analyses showed that the South experienced the steepest increase in mortality for HHD with heart failure, while the Midwest had the steepest rise in HHD without heart failure. By 2030, disparities are projected to widen further, with the South expected to show the greatest increase in both groups. The Northeast had the slowest rise in HHD with heart failure and the smallest projected increase by 2030; this slower rise may reflect regional differences in cardiovascular risk profiles, prevention efforts, or access to hypertension management. Notably, HHD without heart failure in the Northeast was the only subgroup without a statistically significant annual increase. These trends warrant additional investigation to clarify the drivers of regional differences in HHD mortality. However, these regional explanations should be interpreted as speculative, as the present analysis did not directly measure socioeconomic conditions, rurality, or healthcare access.

Despite rising age-related mortality, HHD and heart failure continue to be persistent public health issues in the United States, with heart disease accounting for roughly 25% of all deaths [5,10]. From 1970 to 2022, overall heart disease mortality decreased; however, mortality from specific subtypes, including heart failure and HHD, has reported increases of 146% and 106% [10]. These rising trends have been attributed to factors such as the long-term impact of chronic conditions identified earlier in life, advances in diagnostic screening, and the accumulation of comorbidities [10]. Together, these patterns suggest a potential connection between healthcare access and subsequent mortality outcomes.

The Scorecard on State Health System Performance evaluates each state and its access to care, quality care, efficient use of services, outcomes, income, disparity, and racial equality [10]. According to the 2025 Scorecard, seven of the 12 Midwest states ranked above average, while the remaining five fell below average [11]. In the Northeast, all nine states ranked above average, with five placing in the top 10 nationally [11]. In contrast, only four of the 17 Southern states were rated above average [11]. This aligns with the historically poor cardiovascular outcomes in the South, also referred to as the Stroke Belt States (SBS). These are states whose incidence and fatalities related to strokes are 10% higher than the median rate of all states [12]. However, these differences do not fully explain why our analysis showed the greatest rise in HHD without heart failure in the Midwest.

One possible explanation for the Midwest showing the greatest disparities in HHD without heart failure (ICD-10 I11.9) is the region’s substantial proportion of rural communities [13]. When examining rural-urban differences in health outcomes through the lens of social determinants of health (SDOH), including overall health, clinical care, and health behaviors both cross-sectionally and longitudinally, SDOH were markedly worse in rural communities [13]. Based on 2023 Census data summarized in America’s Health Rankings, the average rural population is 32.0% in the South and 30.1% in the Midwest [14]. This is consistent with the poor health outcomes in the South, displayed by our findings of the steepest increase in HHD with heart failure (ICD-10 I11.0), in addition to the overall highest forecasted gamma regression in the South by 2030. This could also explain the significant rise of HHD without heart failure (ICD-10 I11.9) in the Midwest.

This discrepancy could also be attributed to bias in medical coding. Efforts to uncover bias within professional medical coding have yielded limited evidence, highlighting the importance of continued investigation [15]. Given the rurality and adverse SDOH noted above, it is plausible that coding practices could contribute to higher reported mortality due to HHD without heart failure (ICD-10 I11.9) in the Midwest. Overall, the current literature lacks sufficient data to support the reasoning behind the most significant rise in mortality in HHD without heart failure (ICD-10 I11.9) as seen in our study. Regional differences identified in this analysis should therefore be interpreted as associations rather than causal relationships.

This study has certain limitations. Death certificate coding errors, including misidentification or omission of significant contributing causes, may affect ICD-10 coding and influence the accuracy of results. Regional variation in death certification or ICD-10 coding practices may also contribute to observed differences in mortality patterns. Additionally, the gamma regression model is limited by the relatively short time span included in the analysis and should therefore be viewed as offering general indications of rising mortality rates across regions, rather than precise forecasts. Given the six-year observation window, forecasted values should be interpreted as directional trends rather than precise long-range estimates. Reliance on data from 2018 to 2023 may restrict both the precision and generalizability of long-term projections.

## Conclusions

This analysis demonstrates that a significant rise in mortality from HHD has been observed between 2018 and 2023, with a particularly steep increase in HHD with heart failure in the South and HHD without heart failure in the Midwest. Forecasting analysis suggests that these disparities will continue to expand by 2030. The regional differences observed likely reflect a complex interplay between healthcare access, socioeconomic factors, and systemic disparities. These findings highlight the urgent need for region-specific public health strategies to address these regional disparities.
